# Atomistic polarization model for Raman scattering simulations of large metal tips with atomic-scale protrusions at the tip apex

**DOI:** 10.1515/nanoph-2023-0403

**Published:** 2023-10-20

**Authors:** Jie Cui, Yao Zhang, Zhen-Chao Dong

**Affiliations:** Hefei National Research Center for Physical Sciences at the Microscale and Synergetic Innovation Center of Quantum Information and Quantum Physics, University of Science and Technology of China, Hefei 230026, China; School of Physics and Department of Chemical Physics, University of Science and Technology of China, Hefei 230026, China; Hefei National Laboratory, University of Science and Technology of China, Hefei 230088, China

**Keywords:** tip-enhanced Raman spectroscopy, atomistic polarization model, Raman scattering simulations, sub-wavelength sizes, mental tip, localized surface plasmon

## Abstract

Tip-enhanced Raman spectroscopy (TERS) has recently been developed to push the spatial resolution down to single-chemical-bond scale. The morphology of the scanning tip, especially the atomistic protrusion at the tip apex, plays an essential role in obtaining both high spatial resolution and large field enhancement at the Ångström level. Although it is very difficult to directly characterize the atomistic structures of the tip, the Raman scattering from the apex’s own vibrations of the metal tip can provide valuable information about the stacking of atoms at the tip apex. However, conventional quantum chemistry packages can only simulate the Raman scattering of small metal clusters with few atoms due to huge computational cost, which is not enough since the shaft of the tip behind the apex also makes significant contributions to the polarizabilities of the whole tip. Here we propose an atomistic polarization model to simulate the Raman spectra of large metal tips at subwavelength scales based on the combination of the atomistic discrete dipole approximation model and the density functional theory. The atomistic tip with different sizes and stacking structures is considered in its entirety during the calculation of polarizabilities, and only the vibrational contributions from the tip apex are taken into account to simulate the Raman spectra of the tip. The Raman spectral features are found to be very sensitive to the local constituent element at the tip apex, atomic stacking modes, and shape of the tip apex, which can thus be used as a fingerprint to identify different atomistic structures of the tip apex. Moreover, our approaches can be extended to the metal tips with sub-wavelength sizes, making it possible to consider both the large scale and the atomistic detail of the tip simultaneously. The method presented here can be used as a basic tool to simulate the Raman scattering process of the metal tips and other nanostructures in an economic way, which is beneficial for understanding the roles of atomistic structures in tip- and surface-enhanced spectroscopies.

## 1 Introduction

Tip-enhanced Raman spectroscopy (TERS) technology has become an advanced method in the single-molecule studies due to its high spatial resolution and sensitive chemical recognition abilities [1–8]. Recently, the spatial resolution of single-molecule TERS imaging has reached down to 1.5 Å, realizing the chemical resolution at single-chemical-bond level [5, 6]. Thanks to the atomistic protrusions at the tip apex, the induced plasmonic fields can be localized to the atomistic scale to probe chemical bonds inside a molecule [9–16]. However, considering the large sizes and arbitrary shapes of the metal tips, the atomistic details are usually not taken into account in the classical electrodynamic simulations, which just solve the Maxwell’s equations with smooth boundary conditions [14, 17–24]. Therefore, only those significant changes in the tip morphologies, such as the aperture angles and the apex radii, can be adequately described in these classical methods [24, 25], although the atomistic feature of the tip apex is also phenomenologically introduced as a smooth protrusion in sub-nanometer size during the simulations [13–15]. On the other hand, the experimental Raman spectra for the bare metal tip-substrate systems always exhibit Raman peaks in the low-wavenumber region, which is conjectured to be associated with the vibrations from the atomistic protrusion at the tip apex [26, 27]. Therefore, it is possible to study how atoms stack at the apex of the tips through Raman scattering from the own vibrations of bare metal tips [26–28].

Various methods based on the quantum chemistry calculations, such as the density functional theory (DFT) [29–33] and tight-binding method [34, 35], have been adopted to simulate the polarization behaviors and Raman scattering processes of the metal tips or nanoclusters through the atomistic modeling [27]. For example, the simulated Raman spectra of pure metal clusters based on DFT indicate that the vibrational frequencies and Raman intensities vary considerably with their sizes and atomistic stacking modes [28, 36]. However, due to the huge computational costs, these quantum chemistry calculations are generally limited to the systems with only hundreds of metal atoms or less [37], and the simulations of polarization and Raman scattering of the large metal tip system with millions of atoms are still prohibitively expensive and impractical so far. Therefore, it is very demanding to develop a novel approach to fast simulate the Raman spectra of large-size tips or nanoclusters, even at the sub-wavelength level.

The atomistic discrete-dipole approximation (DDA) method [12, 38, 39] provides an alternative way for calculating the polarization properties without sacrificing the atomistic features of the metal tips. In this model, each metal atom is represented by a polarized dipole induced by the external electric field and the dipole-dipole interactions between them. However, the traditional DDA only deals with determined structures with all the atoms fixed in positions, and the vibrational properties and Raman scattering process are usually not included in this model. Here, we propose a strategy to combine the atomistic DDA method and the DFT calculations together to simulate the plasmonic responses and Raman scattering of the metal tips with sub-wavelength sizes. Inspired by the experiments that the Raman spectrum of the tip might be mainly contributed by the atoms at the apex [26] due to the presence of localized plasmonic “hot spots” [11, 14, 16], only the contribution from those apex atoms is taken into account during the vibrational analysis. The vibrational frequencies and Raman spectral features of the metal tip are found to be very sensitive to the changes in the tip morphologies and atomistic stacking structures. As the height of the tips increases, the plasmonic resonant frequency is red shifted and the Raman spectral features are also influenced, showing different peak intensities although the peak positions are generally unchanged because the same atomistic stacking structures of the tip apexes is adopted for all the tip structures with different heights. On the other hand, if the radius of the tips increases, the Raman spectrum would be changed from discrete peaks to complex envelopes because more atoms at the tip apex are involved in the vibrations. By substituting the apex atom of the tip with different noble metal atoms, the dominant peaks in the corresponding Raman spectra are quite different, providing a very convenient way to judge the type of atom absorbed at the tip apex. Our model can be further extended to simulate the Raman scattering of the metal tips with the length of ∼120 nm at the sub-wavelength scale (>1,000,000 atoms), showing the joint contributions in the polarization process from both the localized plasmon around the tip apex and the propagating plasmon along the sides of the tip. This method provides a convenient way to characterize the plasmonic and vibrational properties of metal tips including both the giant shaft and the atomistic apex, which would be beneficial for understanding the critical role of the tip at the atomistic scale.

## 2 Methods

**Figure 1:**
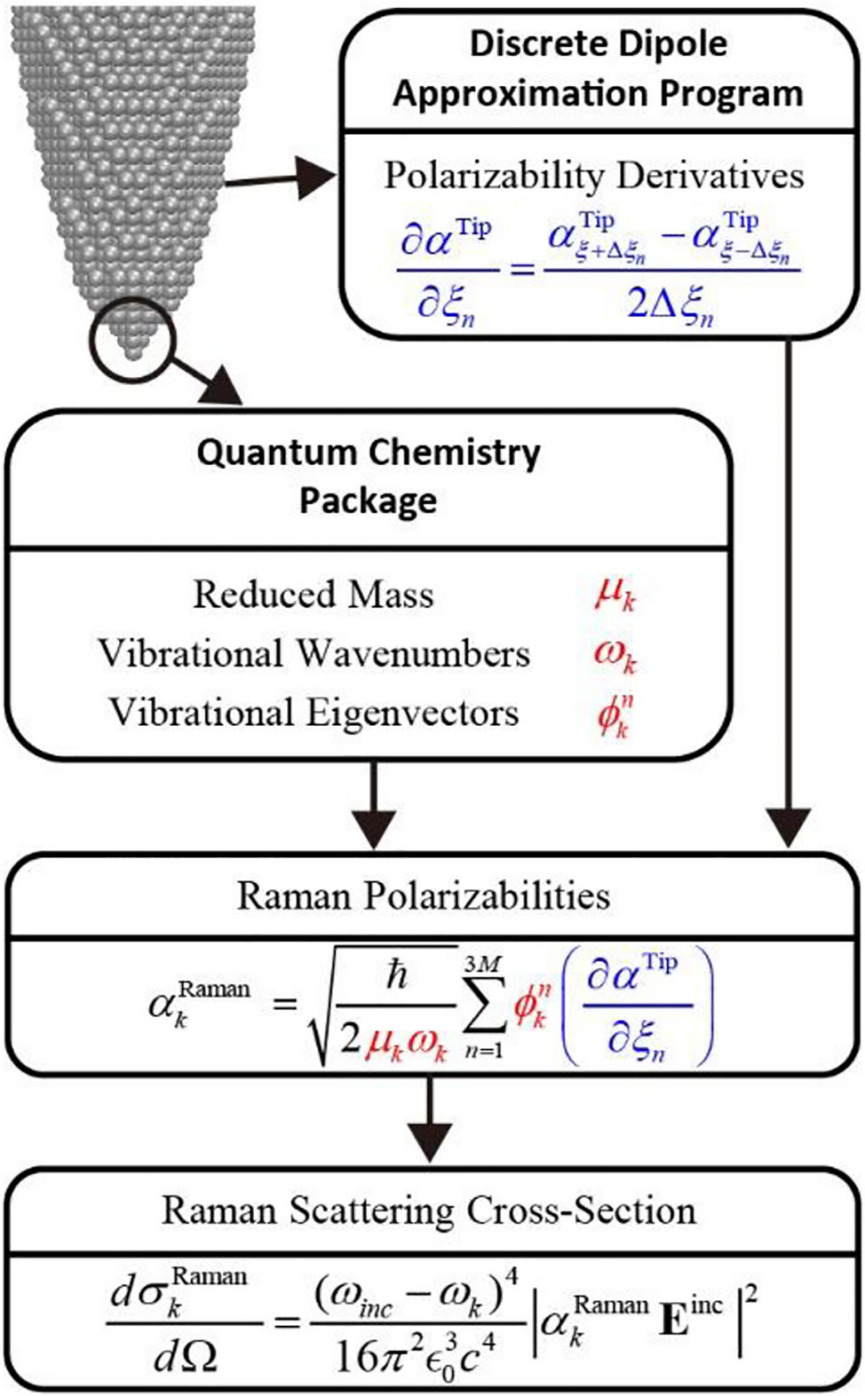
Flow chart for the procedure for atomistic simulation of Raman scattering from the tip.

The full procedure of the atomistic polarization model for simulating the Raman scattering from the atomistic metal tip is shown in Figure 1. Firstly, the derivatives of the polarizability tensors are computed using the atomistic DDA model with the finite difference method [40] by displacing each atom at the tip apex along the *x*, *y* and *z* axes in Cartesian coordinates, respectively. Secondly, a standard vibrational analysis is carried out for the tip apex (the small cluster marked by the circles in Figure 1) by using the quantum chemistry package (e.g., Gaussian16 [41] in our simulations) to obtain the vibrational energies and eigenvectors corresponding to different vibrational modes of the tip apex. By combining the Raman polarizability derivatives and the vibrational eigenvectors calculated above, the Raman tensors corresponding to all vibrational modes of the tip apex can be finally obtained, and the far-field Raman scattering cross-section can be calculated from the radiations of Raman dipole moments induced by the incident electric field for each vibrational mode. The details of this method will be explained as follows.

### 2.1 Atomistic discrete dipole approximation model

In the atomistic DDA model, each atom in a nanocluster is characterized as a point dipole with an isotropic polarizability, and these atoms interact with each other through the dipole-dipole interactions [39]. The atomic dipole moment of each atom is determined by the atomic polarizability and the electric field from both the incident light and the dipolar contributions of other atoms, resulting in the induced dipole moment **p**_*i*_ of the atom *i* expressed as

(1) $$p_{i}=\alpha_{i}\left(E_{i}^{\text{inc}}+\overset{N}{\underset{j\neq i}{\sum }}T_{ij}p_{j}\right),$$

where the first term is ascribed to the response to the incident electric field $E_{i}^{\text{inc}}$ at the *i*th atom’s position, and the second term is ascribed to the response to the other atomic dipole moments. Here ***α***_*i*_ represents the atomic polarizability tensors of atom *i* and **T**_*ij*_ is the dipole–dipole interaction tensor between atomic dipoles *i* and *j*. The above equation can be simplified as a linear set of equations for each atom as

(2) $$\overset{N}{\underset{j=1}{\sum }}A_{ij}\cdot p_{j}=E_{i}^{\text{inc}},$$

where *N* is the total number of metal atoms to compose the tip, and the matrix **A**_*ij*_ is defined as

(3) $$A_{ij}=\left\{\begin{array}{cc} \alpha_{i}^{-1},\; & i=j\\ -T_{ij},\; & i\neq j \end{array}\right..$$

Here the isotropic atomic polarizability tensor only contains diagonal elements from the Clausius–Mossotti relationship **α**_*i*,*ll*_ = (3*V*/4*π*)(*ɛ* − 1)/(*ɛ* + 2), where *V* is the volume of the atom obtained from the lattice constant (e.g., *V* = 17.05 Å^3^ for Ag atom with face-centered cubic structures) and *ε* is the dielectric constant from the experiments [42]. Note that the modified atomic polarizabilities by comparing with the quantum simulations would provide more accurate results [12, 43–45], which would be implemented in our further model. The full expression of the dipole–dipole interaction tensor can be written as

(4) $$\begin{array}{ll} T_{ij} & =-\frac{\exp\left(ikr_{ij}\right)}{r_{ij}}\left[k^{2}\left({\hat{r}}_{ij}{\hat{r}}_{ij}-I\right)+\frac{ikr_{ij}-1}{r_{ij}^{2}}\left(3{\hat{r}}_{ij}{\hat{r}}_{ij}-I\right)\right], \end{array}$$

where *k* is the wavevector of dipole oscillation in free space, *r*_*ij*_ is the distance between atom *i* and *j*, and ${\hat{r}}_{ij}$ is the unit vector for describing these two atoms’ direction. Once the size of the nanoparticles is smaller than ∼1/10 of the wavelength of the incident light, the retardation effect can be neglected and the dipole–dipole interaction tensor can be simplified as $T_{ij}=\left(3{\hat{r}}_{ij}{\hat{r}}_{ij}-I\right)/r_{ij}^{3}$ . In our simulation, the induced atomic dipole moments **p**_*j*_ can be obtained by numerically solving Equation (2) with the precise linear solver from the “linalg” module in Scipy [46] for smaller systems (<20,000 atoms). For the large-size tips with about one million atoms, the sparse matrix is introduced and the GMRES solver [47] is used.

### 2.2 Plasmonic response of a metal tip

After obtaining the values of atomic dipole moments (**p**_*i*_), the far-field and near-field properties of the metal tip can be directly obtained as a sum of the contributions from each atom. In particular, the far-field absorption cross section can be expressed as

(5) $$\sigma_{\text{abs}}=\frac{4\pi \omega }{c}Im\left\{\overset{N}{\underset{i=1}{\sum }}p_{i}\cdot E^{\text{inc}}\right\}/{\left|E^{\text{inc}}\right|}^{2},$$

where *ω* is the frequency of the incident field, and *c* is the speed of light. The near-field spatial distribution can be also evaluated from the sum of the atomic contributions as

(6) $$E^{\text{loc}}\left(r\right)=\overset{N}{\underset{i=1}{\sum }}\overset{↔}{G}\left(r,r_{i}\right)p_{i},$$

where the near-field Green function $\overset{↔}{G}\left(r,r_{i}\right)$ between the dipole source position **r**_*i*_ and the local field position **r** can be written as

(7) $$\overset{↔}{G}\left(r,r_{i}\right)=\frac{1}{{\left|r-r_{i}\right|}^{3}}\left(\frac{3\left(r-r_{i}\right)\left(r-r_{i}\right)}{{\left|r-r_{i}\right|}^{2}}-I\right).$$

### 2.3 Simulation of the Raman scattering from the tip apex

For a given structure of the metal tip, its polarizability can be evaluated from the induced dipole moments of the tip according to the polarized electric field along *x*, *y* and *z* directions as

(8) $$\begin{array}{ll} \alpha^{\text{Tip}} & =\left[\begin{array}{ccc} \alpha_{xx} & \alpha_{xy} & \alpha_{xz}\\ \alpha_{yx} & \alpha_{yy} & \alpha_{yz}\\ \alpha_{zx} & \alpha_{zy} & \alpha_{zz} \end{array}\right]\\ & =\left[\begin{array}{ccc} P_{E_{x}}^{\text{Tip}}/\left|E_{x}\right| & P_{E_{y}}^{\text{Tip}}/\left|E_{y}\right| & P_{E_{z}}^{\text{Tip}}/\left|E_{z}\right| \end{array}\right], \end{array}$$

where $P_{E_{x}}^{\text{Tip}}=\sum_{i=1}^{N}p_{i}$ is the total dipole moment of the tip induced by the *x*-polarized incident electric field **E**_*x*_. Since the Raman intensity is directly related to the polarizability derivatives, the values of the derivatives with respect to each atomic displacement are calculated by the finite difference method [40] through the repetitive DDA simulations with the coordinate displacement Δξ=0.01 Å. Here the index *ξ* represents the *x*, *y* or *z* coordinates of the equilibrium structure. Hence, one can express the polarizability derivative corresponding to the *n*th atomic coordinate as

(9) $$\frac{\partial \alpha^{\text{Tip}}}{\partial \xi_{n}}=\frac{\alpha_{\xi +\Delta \xi_{n}}^{\text{Tip}}-\alpha_{\xi -\Delta \xi_{n}}^{\text{Tip}}}{2\Delta \xi_{n}},$$

where $\alpha_{\xi \pm \Delta \xi_{n}}^{\text{Tip}}$ is the polarizability tensor of the tip with the deformed structure *ξ* ± Δ*ξ*_*n*_. The Raman polarizability can be expressed through a coordinate transformation from the normal coordinates *Q*_*k*_ to the atomic coordinates *ξ*_*n*_ as

(10) $$\alpha_{k}^{\text{Raman}}=\left(\frac{\partial \alpha^{\text{Tip}}}{\partial Q_{k}}\right)Q_{k}=\sqrt{\frac{\hbar }{2\mu_{k}\omega_{k}}}\overset{M}{\underset{n=1}{\sum }}\phi_{k}^{n}\left(\frac{\partial \alpha^{\text{Tip}}}{\partial \xi_{n}}\right),$$

where *μ*_*k*_ and *ω*_*k*_ denote the reduced mass and frequency of the *k*th vibrational mode, $\phi_{k}^{n}$ denotes the vibrational eigenvector corresponding to the *k*th vibrational mode. The total number of the atomic coordinate is *M* = 3 × *N*. After applying an incident electric field **E** ^inc^, the induce Raman dipole moment for the *k*th vibrational mode can be obtained as $p_{k}^{\text{Raman }}=\alpha_{k}^{\text{Raman}}E^{\text{inc }}$ . As a result, the differential Raman scattering cross section of the tip can be calculated from

(11) $$\frac{d\sigma_{k}^{\text{Raman }}}{d\Omega }=\frac{\omega_{\text{R}}^{4}}{16\pi^{2}\varepsilon_{0}^{3}c^{4}}{\left|\alpha_{k}^{\text{Raman }}E^{\text{inc }}\right|}^{2},$$

where the radiation frequency of Raman dipole moment for the *k*th vibrational mode can be expressed as *ω*_*R*_ = *ω*_inc_ − *ω*_*k*_, and *ω*_inc_ is the frequency of incident light.

In our simulations, only the perturbed displacements of the atoms at the tip apex are considered during the calculations of the polarizability derivatives ∂*α* ^Tip^/∂*ξ*_*n*_. The values of polarizability derivatives can be also directly evaluated from the entire deformation of the tip apex following the corresponding vibrational eigenvectors without the coordinate transformation shown in Equation (10), which would result in the same derivative values if the incident light is plane wave [48]. The vibrational analysis of the tip including the vibrational eigenvector $\phi_{k}^{n}$ , vibrational frequency *ω*_*k*_, and the reduced mass *μ*_*k*_ can be calculated by using the quantum chemistry [49] or molecular dynamics packages [50]. Here, we use the Gaussian16 package with the hybrid functional PBE0 and the def2-SVP basis set for the vibrational analysis simulations [41].

## 3 Results and discussions

### 3.1 Influence of atomistic changes in tip apex

**Figure 2:**
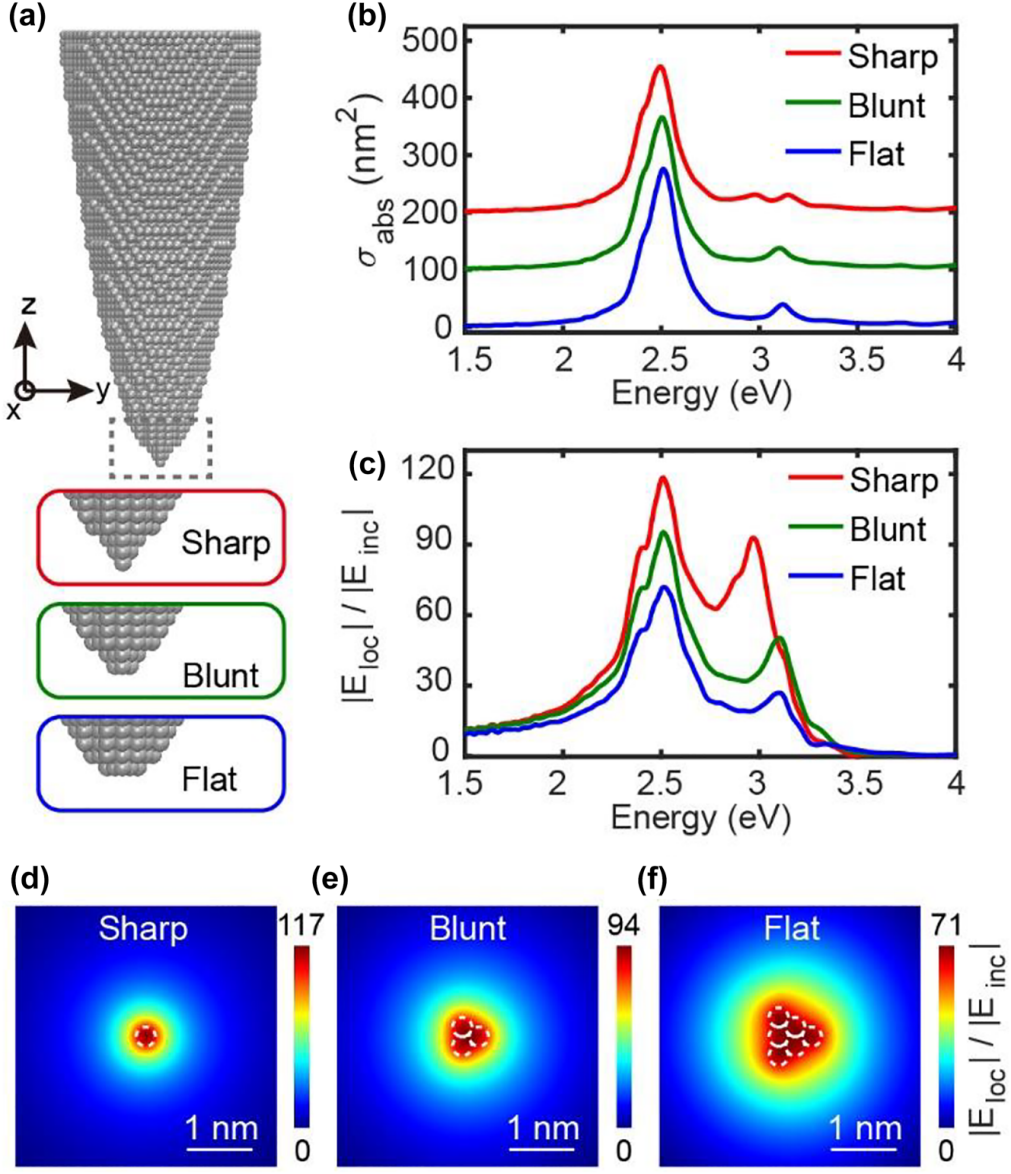
Plasmonic response properties of Ag tips. (a) Atomistic structures of Ag tips with three different atomic-scale apexes. (b–c) Absorption and near-field enhancement spectra of the three tips for an incident planewave with the electric field polarized along the *z*-axis. (d–f) Induced local electric field enhancement distribution in the *xy*-plane at a distance of 0.2 nm from the apex surface for an incident optical planewave with the energy of 2.5 eV. The white dotted circles with a radius of 0.144 nm represent the outlines of atoms at the tip apexes.

As shown in Figure 2(a), a silver cluster with a parabolic shape and a height of 15.2 nm is built up as the model system of the tip by stacking 16,512 atoms with a crystal lattice constant of 4.0897 Å [51], and three types of atomistic changes in tip apexes, namely, the sharp tip (intact Ag_16512_ cluster), the blunt tip (Ag_16511_ cluster by removing the apex atom from the sharp tip), and the flat tip (Ag_16508_ cluster built up by removing two layers of apex atoms from the sharp tip) are considered to study the influence of apex morphologies. Figure 2(b) shows far-field absorption spectra for these tips when the electric field of the incident planewave is polarized along the *z*-axis, showing similar spectral features with a main peak located at ∼2.5 eV. This resonant peak can be assigned to the dipolar plasmon (DP) mode according to the surface charge distribution around the tip (see Supplementary Figure 1). Two additional resonant peaks with higher energies at ∼3.1 eV are also observed, which are associated with quadrupolar plasmons (QP), which split into two peaks for the sharp tip with the atomic protrusion at the apex. The DP and QP peaks can also be identified in the near-field spectra of the local field enhancement under the tip apex (as shown in Figure 2(c)), showing stronger intensities of the near fields for the sharp tip. Such results imply that the near-field response of the tip would be very sensitive to the atomistic changes at the tip apex. Moreover, the spatial distributions of the local plasmonic field can directly reflect the outlines of atomic stacking patterns at the tip apex. Figure 2(d)–(f) show the corresponding local electric field distributions for the *xy*-plane with a distance of 0.2 nm from the surface of the apex atom in resonant condition with the DP mode (*ω* = 2.51 eV) for different tips. The local electric fields are generally confined at the atomic scale near the tip apex, exhibiting great field enhancement under the tip. For the sharp tip with only one atom at the tip apex, the local electric field distribution shows a circular shape, while for the blunt and flat tips, triangular shapes appear because of the truncated pyramid structures at the tip apexes. Such feature will be lost at longer distances (typically larger than 4–6 Å, see Supplementary Figure 2), showing a blurred round shape without any atomistic details [52, 53].

### 3.2 Vibrational modes of Ag tip apex

**Figure 3:**
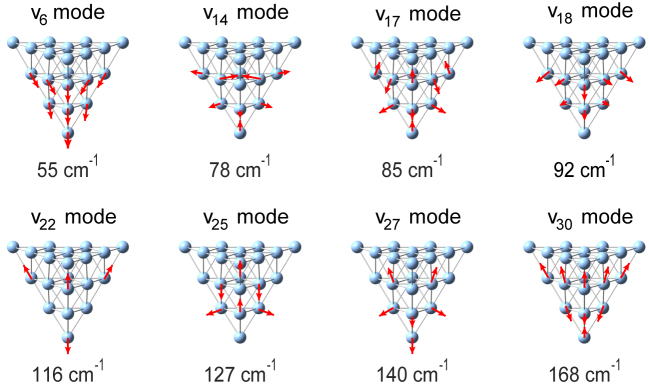
Atomic motions for eight dominant vibrational modes of the tip apex (a Ag_20_ cluster). The red arrows highlight the directions and amplitudes corresponding to the characteristic atomic displacements.

Since the incident light has been strongly confined to the tip apex region, the Raman scattering signals would be mainly contributed by the vibrations of the apex atoms rather than the rest part of the tip. Therefore, we first performed vibration analysis of a Ag_20_ cluster to mimic the tip apex through the DFT calculations with Gaussian 16 package [41]. Based on the experiences in previous reports [27, 54, 55], three layers containing ten silver atoms at the tip apex are relaxed for optimization and the other atoms are fixed during the calculations to save the computing time. There are thirty vibrational modes in total, and all the vibrational frequencies are below 200 cm^−1^. Figure 3 presents eight dominant vibrational modes, with the schematics of the displacement of the atoms for each mode demonstrated by the red arrows. The vibrational mode with the energy of 55 cm^−1^ corresponds to the collectively out-of-plane vibrations of all atoms at the tip apex. The vibrational mode with the energy of 78 cm^−1^ corresponds to the relative vibration of the apex atom and its nearest neighbor atom along the tip axis while other atoms exhibit approximate in-plane vibrations. The vibrational mode with the energy of 140 cm^−1^ corresponding to *v*_27_ mode represents the so-called breathing mode, which involves collective vibration of the four atoms at the tip apex. The complete and detailed identification of the vibrational modes of the tip apex is shown in Supplementary Video 1.

**Supplementary Video 1:** 

### 3.3 Influence of tip shapes on the Raman scattering from tip apex

**Figure 4:**
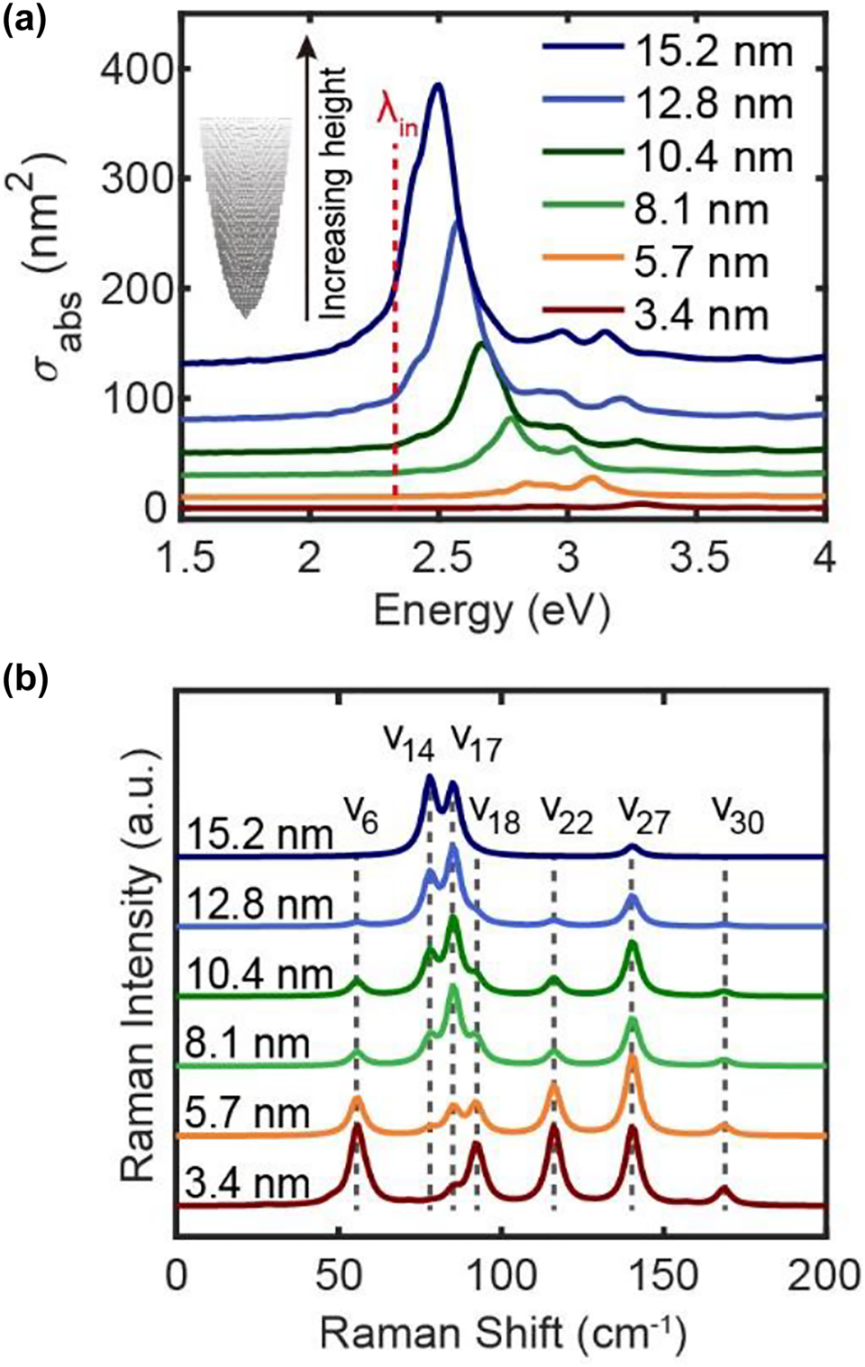
Absorption and Raman spectra of the tips with different heights. (a) Absorption spectra and (b) normalized Raman spectra of the tips with the shape of a paraboloid and the different heights from 3.4 nm to 15.2 nm. The stacking structure of the tip apex is similar to that shown in Figure 3. The red dashed line in (a) represents the frequency of the incident electric field at 2.33 eV for Raman scattering simulations in (b).

In the above vibrational analysis, the Raman scattering process can also be discussed once the polarizability derivatives are also calculated in DFT simulations. However, such simulation can only be carried out for the small clusters with hundreds of metal atoms. On the other hand, the polarization behavior of the metal tip involves the contributions in its entirety, and it would be far from the truth if only the polarizabilities from the apex atoms are considered. Moreover, the plasmonic response of the tip would be also strongly dependent on the shapes and sizes, requiring to take the whole part of the tip into account. Figure 4 shows the size-dependent plasmon response and Raman scattering of different Ag tips with the same apex radius (*R* = 0.8 nm) but different heights (from ∼3.4 nm with 725 atoms to ∼15.2 nm with 16,512 atoms). The shaft of the tip is built through the Ag crystal structure (with the *z*-axis along the [111] direction) and the optimized Ag_20_ structure as mentioned in Figure 3 is epitaxially attached to the shaft as the tip apex. The incident electric field is a planewave with a wavelength of 532 nm and is polarized along the *z*-axis. As shown in Figure 4(a), the plasmonic resonance frequencies are generally red shifted as the tip height increases. For example, the DP mode located at 2.8 eV for the short tip with the height of 3.4 nm is shifted to 2.5 eV once the tip height increases to 15.2 nm, which is similar to the red-shift trend of the longitudinal dipolar mode of the nanorod with increasing length [44, 56]. The absolute intensities of absorption cross sections are increased with the increasing size of the tip, and the QD as well as other higher-order modes are also red shifted in general.

The simulated Raman spectra of tips with different heights are shown in Figure 4(b), which are normalized to show the differences in relative intensities between different vibrational modes. It should be noted that although only the vibrations from the last three layers of apex atoms are considered, the polarizability of the entire tip as well as its derivatives corresponding to these vibrational modes always take all the atoms into account. The influence of apex atom numbers on the vibrational modes is detailly discussed in Supplementary Section 4. Seven dominant Raman peaks appear in the spectra, showing different relative intensities for different tip sizes. As the height of the tip increases from 3.4 nm to 15.2 nm, the relative intensities of *v*_14_ and *v*_17_ peaks increase, while those of *v*_6_, *v*_18_, *v*_22_, *v*_27_ and *v*_30_ modes decrease gradually. There might be two reasons that account for the changes in spectral features for different tips: One is the red-shift of the DP mode, resulting in different enhancement factors for different vibrational modes; the other is the differences in the total polarizabilities of the tip, resulting in the differences in the equivalent polarizability as well as its derivatives of each atom. As a result, only *v*_14_, *v*_17_ and *v*_27_ modes are obvious and dominant in the Raman spectrum of longer tips.

**Figure 5:**
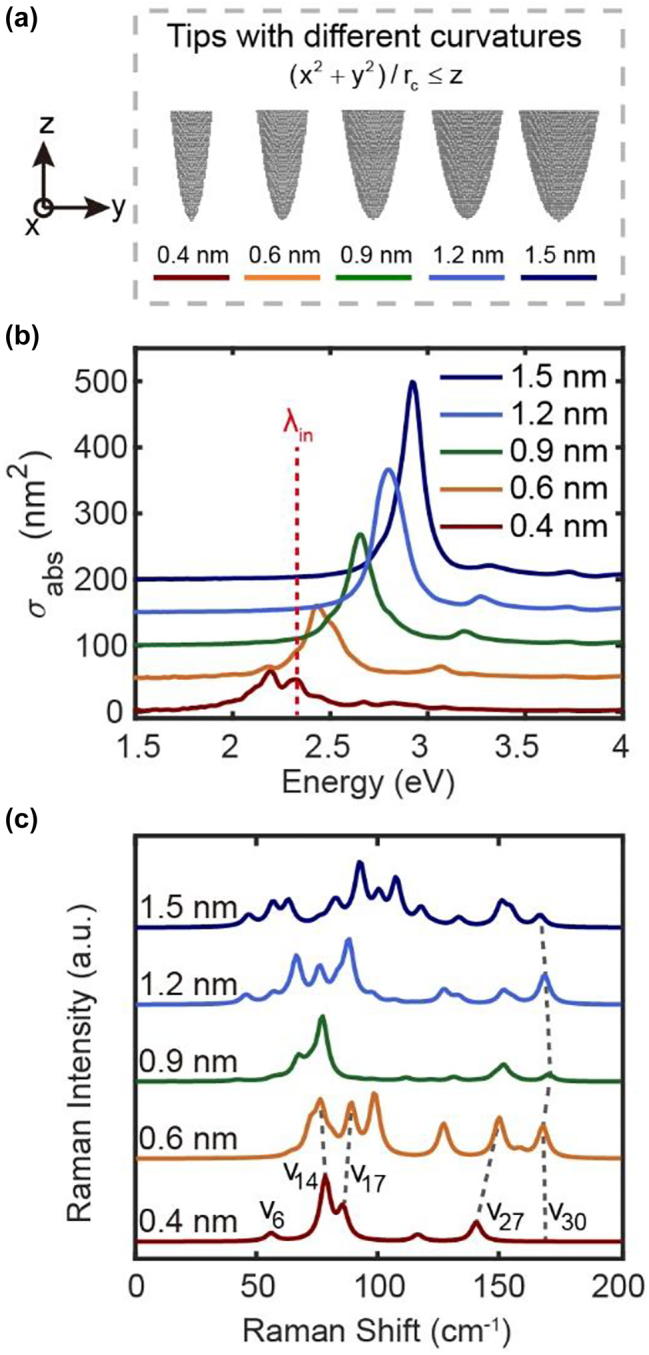
Absorption and Raman spectra of the tips with different radii. (a) The tip structures with the different curvature radii from 0.4 nm to 1.5 nm. (b) Absorption spectra and (c) normalized Raman spectra corresponding to different tip structures in (a). The detailed stacking structures of tip apexes are shown in Supplementary Figure 4. The red dashed line in (b) represents the energy of the incident electric field at 2.33 eV for Raman scattering simulations in (c).

Similar results can be also observed for the absorption and Raman scattering spectra of the tips with the same height (∼11.6 nm) but different tip radii (from 0.4 nm to 1.5 nm as shown in Figure 5(a)). Once the tip radius is changed, the stacking structures and the vibrational modes of apex atoms would be also influenced, which are detailed analyzed in Supplementary Figure 4 and Video 2. As shown in Figure 5(b), in contrast to the tip height changes, the main DP peaks of different tips in the far-field absorption spectra are blue-shifted as the apex radius increases. A split of the DP peak at *ω* = 2.20 eV can be observed for the slim tip with the radius *R* = 0.4 nm, since the atomistic roughness is more prevalent in such a tip structure to make the assignment of the plasmonic modes more difficult (see Supplementary Figure 5 for details). Figure 5(c) shows the Raman spectra corresponding to these tips, from which it can be seen that as the radius of the tip increases, the feature of Raman spectrum changes from individual narrow peaks to complex wide envelopes, and the relative intensities of the Raman peaks also vary significantly. Different from the situation in changing the tip heights where the apex structures are always the same, the atomic stacking structures at the apex of the tips with different radii would be quite different. Usually, the blunter tip would contain more atoms at the apex (assuming that only the first three atomic layers of the apex are considered in all tips), resulting in more vibrational modes with similar frequencies as well as the overlapping in Raman spectra. On the contrary, the Raman spectrum of the sharper tip is contributed by the vibrations of fewer atoms at the apex, resulting in the discrete Raman peaks with fewer overlaps. In this sense, it is difficult to assign the same vibrational modes for these tips with different radii, and we could only identify very few modes that always exist in all tips. For example, the Raman peak at ∼170 cm^−1^ related to the vibrational mode *v*_30_ of the apex atoms is always visible for both the sharp or blunt tips. Moreover, if the tip radius is much larger (e.g., >10 nm), there always exist atomistic protrusions or local structures at the surface or very apex of the tip [13, 26], thus the final spectra would be mainly determined by the vibrations of such atomic details or roughness rather than the whole large tip.

**Supplementary Video 2:** 

### 3.4 Influence of apex-atom substitution on the Raman scattering from tip apex

**Figure 6:**
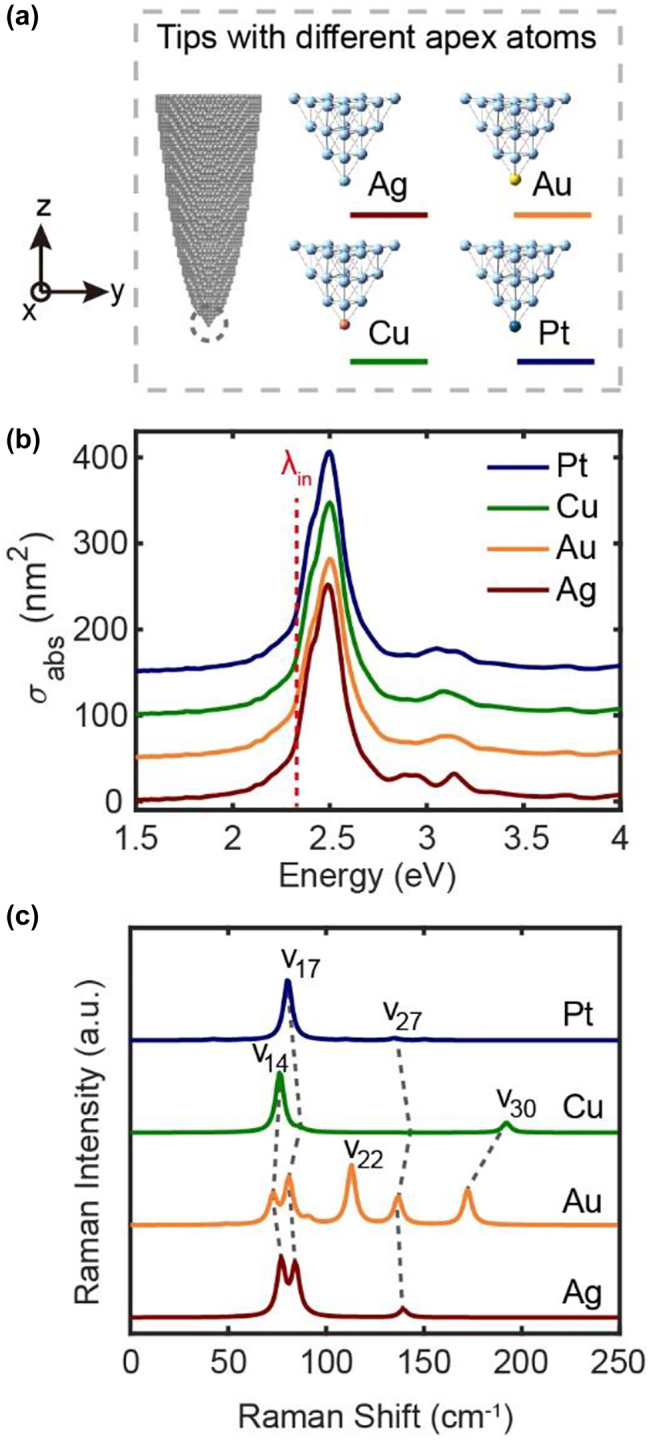
Absorption and Raman spectra of the tips with different apex-atom substitutions. (a) The tip structures with the different noble mental atoms adsorbed at the tip apex. (b) Absorption spectra and (c) normalized Raman spectra corresponding to those tips in (a). The red dashed line in (b) represents the energy of the incident electric field at 2.33 eV for Raman scattering simulations in (c).

In the above discussions, we have found that both the far-field absorption spectra and the near-field Raman spectra of the tip apexes are very sensitive to the atomistic changes of the tip structures. This raises an interesting question that whether it is possible to characterize the single-atom changes of the tip (e.g., with only one noble metal atom is substituted at the apex). Here we first simulate the plasmon response and Raman spectra for tips with different substitutions of noble metal atoms (e.g., a Au, Cu or Pt atom at the apex). The optimized geometries of the apex clusters corresponding to these tips are shown in Figure 6(a). No observable changes can be observed in the far-field absorption spectra in Figure 6(b) by only replacing one single atom of the apex. However, the simulated Raman spectra corresponding to these tips do exhibit some differences. For example, the Raman spectrum of the Ag tip with a single Au atom substitution exhibits two obvious peaks related to *v*_22_ and *v*_30_ modes, which are generally very weak for the pure Ag tip. For the Cu-adsorbed Ag tip, the *v*_30_ mode is also visible in the simulated spectrum but greatly blue-shifted to ∼193 cm^−1^, while the intensity of *v*_17_ peak becomes very weak. For the Pt-adsorbed Ag tip, there is only one dominant peak corresponding to *v*_17_ mode, as well as a very weak *v*_27_ peak to be observed. Considering the similarities in all vibrational eigenvectors for the tips with different substitution atoms (Supplementary Video 3), the differences in the spectral features can be attributed to interactions between the apex atom and its neighboring atoms because of the changes in atomic polarizabilities. Therefore, it would be very convenient to judge the type of atom adsorbed at the tip apex directly from the distinguishable fingerprints in the Raman spectral features.

**Supplementary Video 3:** 

### 3.5 Raman spectra of the tip with sub-wavelength size

**Figure 7:**
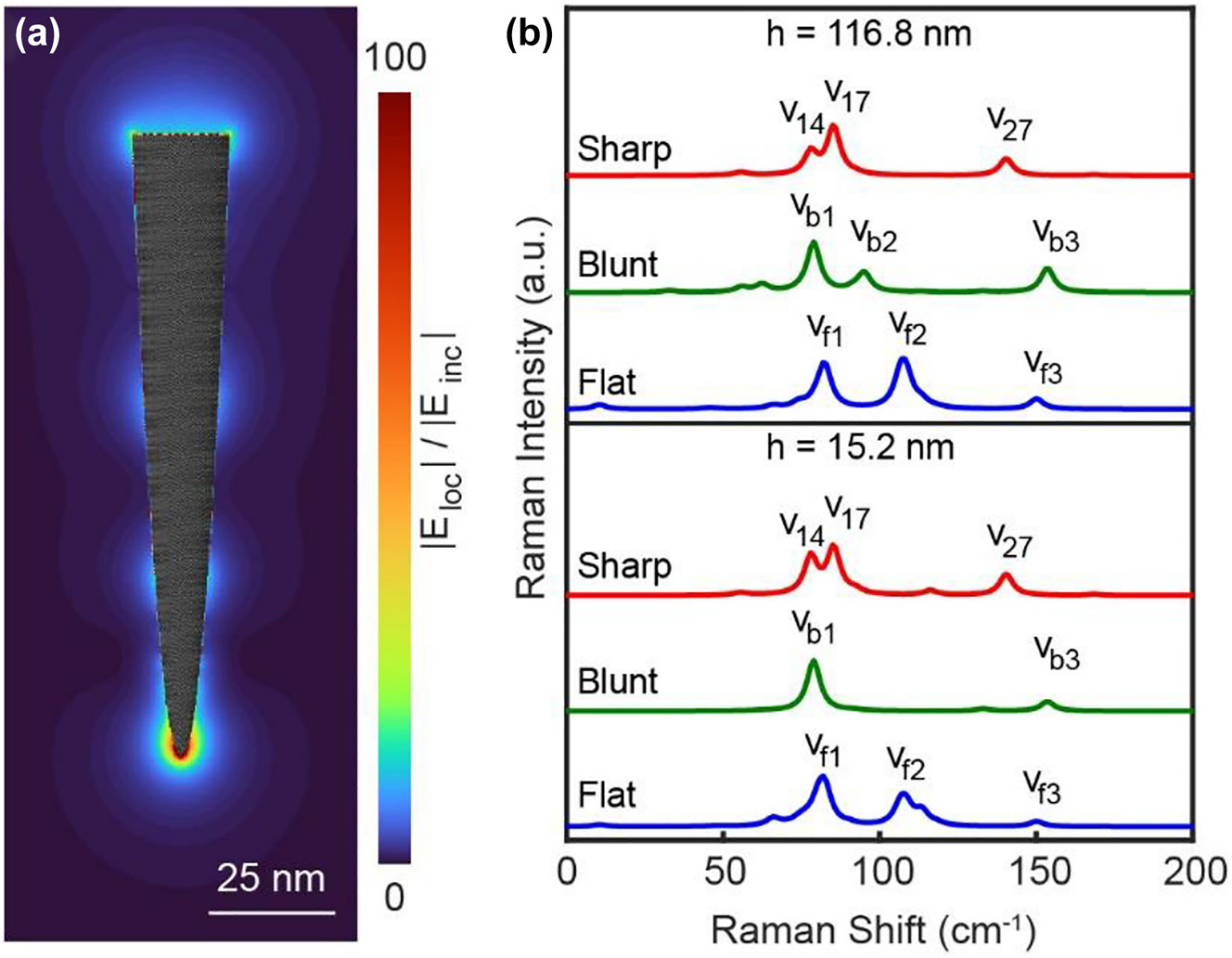
Local electric field distribution and Raman spectra of the tip with sub-wavelength size. (a) Induced local electric field distribution of a large-size tip composed of 1,002,729 atoms and with a height of ∼116.8 nm. (b) Normalized Raman spectra of the tips with a height of ∼116.8 nm (top) and ∼15.2 nm (bottom), respectively with three different apex morphologies (same as Figure 2(a)). The stacking structures and the dominant vibrational modes of the tip apexes are shown in Supplementary Video 4.

The sizes of the tips discussed above are generally much smaller than the wavelength of the incident light, although there are already >10,000 atoms involved. In order to verify whether our method can be applied to the tip with a sub-wavelength size, we construct a parabolic tip composed of 1,002,729 atoms with the height of ∼116.8 nm and the tip radius of 0.8 nm (see Figure 7(a)). For such a large-scale tip, a full description of the interaction matrix is inefficient and unnecessary. Considering that the interaction between atomic dipoles decays very fast following the *r* ^−3^ law, the sparse matrix for **T** is adopted with variable cut-off values for atoms far from the tip apex adapted for the matrix elements following the function $T_{c}=10^{-6}\times 10^{\left(L-3\right)/L_{r}}$ Å^−3^, where *L* represents the layer index where the atom is located and increases for atoms from the tip apex (*L* = 1) to the tip shaft, and *L*_*r*_ represents the rate parameter to tune the cut-off values and is set to be 30 in our simulations (see the detailed discussion in Supplementary Section 6). The minimum and maximum values of *T*_*c*_ are set as 10^−6^ Å^−3^ and 10^−4^ Å^−3^ for the atoms very near or very far from the tip apex, respectively. The retardation effect [19, 57] is considered during the calculation of the interaction matrix, and the GMRES solver [47] is adopted to solve a set of linear response equations self-consistently because of its support for the sparse matrix. The induced local electric field distribution under an incident planewave with a wavelength of 532 nm and the polarized direction along the tip axis is shown in Figure 7(a). Although the maximum local electric field distribution is still in the proximity of the tip apex, the propagating plasmon along the sides of the tip can be clearly observed, resulting in the joint contributions in the polarization process from both the localized and propagation plasmons (see Supplementary Section 7 for the comparison with the simulated results using the continuum method). Figure 7(b) shows the normalized Raman spectra of tips with three different apex morphologies (same as Figure 2(a)) corresponding to two different heights of 15.2 nm and 116.8 nm, respectively. Both the small and large tips show similar spectral features, further confirming that the Raman spectral features are mainly determined by the tip apex and the influence of the tip shaft would be similar as long as the tip size is large enough. Especially, for the sharp tip, the Raman spectra are dominated by *v*_14_, *v*_17_ and *v*_27_ modes, which are consistent with the Raman spectra shown in Figure 4(b). For the blunt tip, the Raman spectra are dominated by *v*_b1_ mode at 78 cm^−1^ and *v*_b3_ mode at 153 cm^−1^, which are similar to *v*_14_ and *v*_30_ modes of the sharp tip. For the flat tip, the Raman spectra are dominated by *v*_f1_, *v*_f2_ and *v*_f3_ modes at 82 cm^−1^, 106 cm^−1^, and 152 cm^−1^, respectively. The detailed assignment and the schematics of atomic displacements for the dominant vibrational modes are shown in Supplementary Video 4. Therefore, for the simulation of Raman spectra of the tip, it is generally sufficient by modeling a short tip with the height of tens of nanometers to mimic the real tip systems.

**Supplementary Video 4:** 

## 4 Conclusions

In conclusion, we have proposed a method by combining the DDA method and the DFT calculations together to simulate the Raman scattering from the apex of metal tips as a general atomistic approach. The derivatives of the polarizability related to the atomic displacement are constructed using DDA within the finite difference regime, and each atom of the tip is endowed with a dipole moment determined by solving the response equations. The vibrational eigenvectors and frequencies of the tip apex are obtained by applying the vibrational analysis using DFT calculation. The Raman signals from the tips are subjected to the combined influences of these two parts of calculations. The simulated Raman spectra were found to be very sensitive to the morphology (height, apex radius, etc.) and atomic substitution of the tip apex, and thus can be used as a fingerprint to identify different atomistic structures of the tip. Moreover, by assuming a cut-off condition for the interaction and adopting the sparse matrix for linear solving, we can push the number of atoms of the tip to more than 1 million atoms during the Raman scattering simulations, making it possible to consider both the large size and the atomistic details of the tip simultaneously. Our method presented here may provide a strategy to simulate the Raman scattering process of the metal tips or other nanostructures in an economic way, which can be used as a basic tool to study and understand the roles of atomistic structures in tip- and surface-enhanced spectroscopies.

## Supplementary Material

Supplementary Material Details
