# Supplementary material for: Atomistic polarization model for Raman scattering simulations of large metal tips with atomic-scale protrusions at the tip apex
Source: Nanophotonics. 2023 Oct 20;12(21):4031–42. doi: 10.1515/nanoph-2023-0403 (PMC11501578; doi:10.1515/nanoph-2023-0403)
Supplement: Supplementary file 9 — Supplementary Material Details [file j_nanoph-2023-0403_suppl_001.pdf]

Supplementary Material for

Atomistic Polarization Model for Raman Scattering Simulations of Large Metal Tips with Atomic-Scale Protrusions at the Tip Apex

Jie Cui[1], *Yao Zhang*[1,2,3*], *Zhen-Chao Dong*[1,2,3*]

[1]Hefei National Research Center for Physical Sciences at the Microscale and Synergetic Innovation Center of Quantum Information and Quantum Physics, University of Science and Technology of China, Hefei, 230026, China.

[2]School of Physics and Department of Chemical Physics, University of Science and Technology of China, Hefei, 230026, China.

[3]Hefei National Laboratory, University of Science and Technology of China, Hefei, 230088, China.

[*]Emails: zhy2008@ustc.edu.cn; zcdong@ustc.edu.cn

S1. Construction of the tip geometry

The surface of the tip is assumed to be parabolic in shape, and the geometric configuration of the tip can be obtained by cutting a bulk silver crystal with specific boundary conditions. The detailed construction of the geometry for the tip involves three steps: Firstly, we constructed a rectangular-shaped Ag crystal with dimensions larger than the tip according to the crystal structure of silver with the lattice constant of 4.0897 Å. The <111> direction of this crystal is defined as the z-axis. Secondly, the boundary conditions described by the parabolic surface is applied to construct the tip. By judging each atom's position and remove those atoms outside the boundary, we can construct the geometry of the tip with atomic details. Finally, to obtain a relaxed geometry of the tip apex, DFT calculations are performed to optimize the positions of atoms within the three layers from the tip apex. The rest atoms are kept frozen during all the optimization and vibrational analysis processes.

S2. The assignment of the plasmon modes of different Ag tips

In order to assign the plasmon modes of the tips corresponding to the resonant peak in the simulated absorption spectra, we introduce the concept of equivalent surface charge distribution to demonstrate the differences of plasmonic responses under different frequencies of incident light. Since the induced atomic dipole moments have been obtained as the solution of the atomistic DDA model, we can convert the atomic dipole $\mathbf{p}_n$ to a spatial distribution of charges $Q(\mathbf{r})$ through the following function as

$$Q(\mathbf{r}) = -\sum_{n=1}^{N} \mathbf{p}_n \cdot \nabla G_n(\mathbf{r}) \,, \qquad (1)$$

where N is the number of atoms of the tip, $\mathbf{p}_n$ is induced dipole moment of the atom n, and $G_n(\mathbf{r}) = \left(1/\pi^{3/2}\alpha_n^3\right)e^{-(\mathbf{r}-\mathbf{r}_n)^2/\alpha_n^2}$ is a 3D Gaussian function centered at atomic position $\mathbf{r}_n$ with a width determined by α_n (which is adopted as the van der Waals radius of Ag atom of 1.72 Å in our simulations). In this way, the surface charge distribution of the tip can be obtained through a summation of the contributions from all induced atomic dipole moments projected on the van der Waals surface.

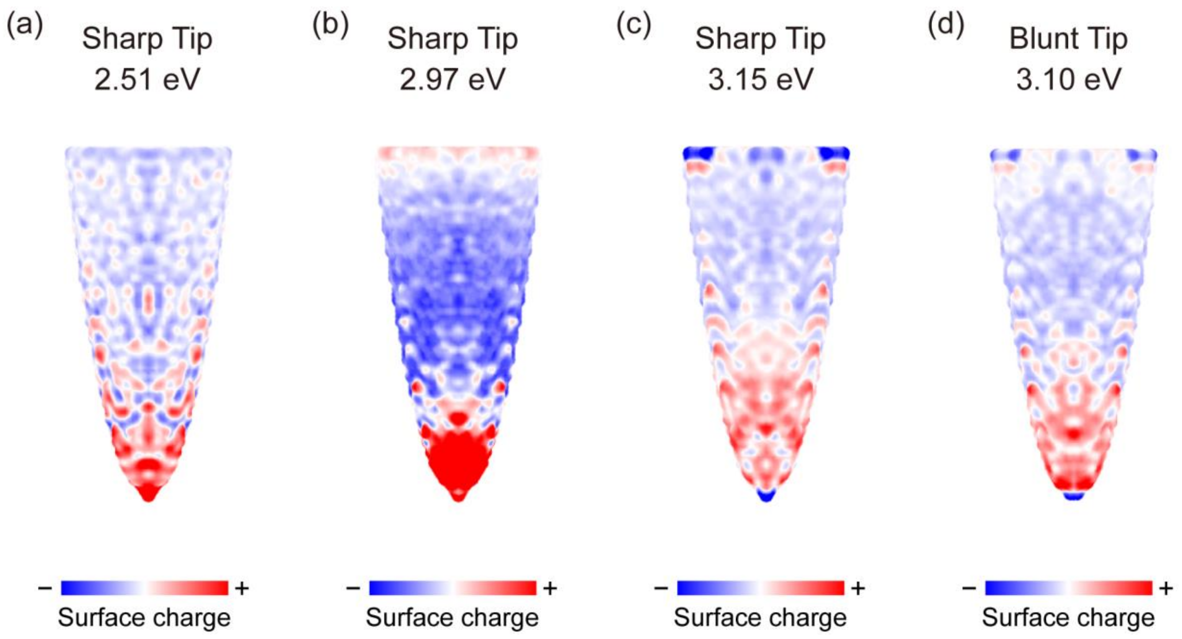

Figure 1. (a-c) The surface charge distribution for the sharp tip corresponding to the plasmon modes at 2.51 eV, 2.97 eV and 3.15 eV and (d) for the blunt tip at 3.10 eV. The tip height is 15.2 nm and the apex radius is 0.8 nm. The charge values are projected on the van der Waals surface of the tips with the isovalue of 3 Å. The maximum/minimum surface charges are resaled to amplify the surface charge distribution features.

S3. The local electric field distribution at different distances from the tip apex

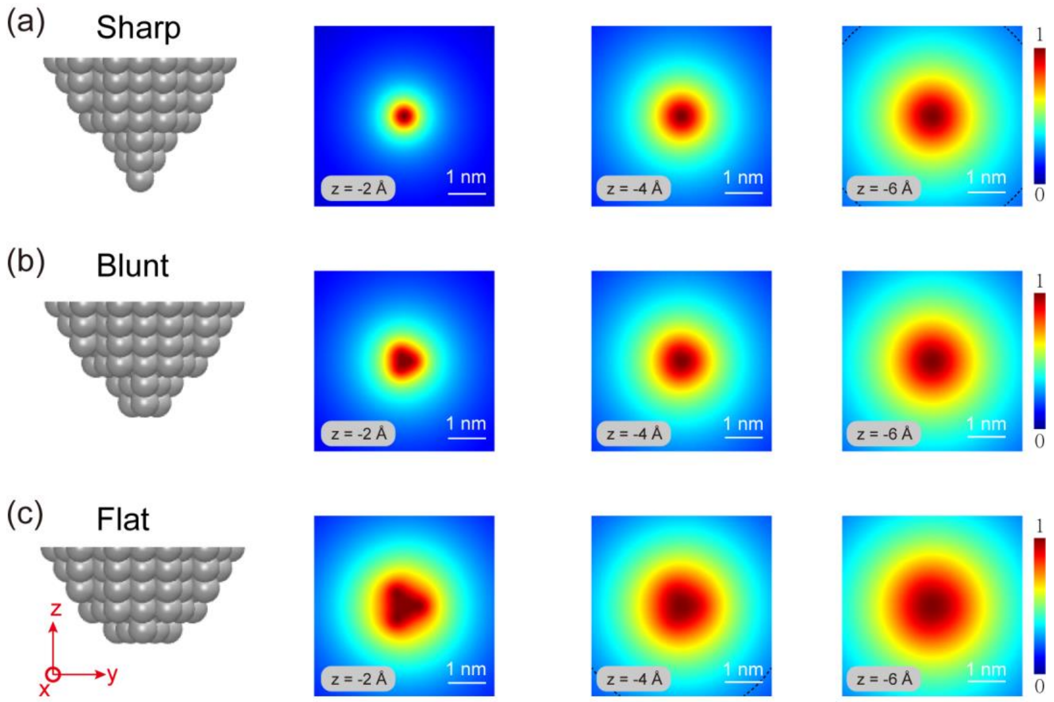

Figure 2. Normalized local electric field enhancement distribution for the sharp tip (a), the blunt tip (b) and the flat tip (c) in the *xy*-plane at a distance of 0.2 nm, 0.4 nm and 0.6 nm from the apex surface of the tip with a height of 15.2 nm, respectively. The incident plane wave is at a resonant mode with the energy of 2.5 eV.

S4. The influence of apex atom numbers on the vibrational modes of the tip

In our model, only the first three layers of tip apex atoms are considered during the vibrational analysis of the tip because of the following reasons: From the experimental results, the Raman spectral features would be significantly changed by just modifying the apex morphologies at the atomistic scale [1]; From the theoretical simulations, the small size of the “hot spot” at the tip apex demonstrated (e.g., “picocavity” in atomic scale [2-4]) would result in the Raman response of the tip to be more sensitive to the displacement of the apex atoms. However, it is really difficult to

perform a through analysis to determine how many atoms at the apex are involved in the tip vibrations due to the limitations in theoretical simulations and experimental measurements. Moreover, larger clusters would require more expensive calculations for structure optimization and vibration analysis. Therefore, only the first three layers of a Ag_{20} cluster are chosen and set free for the vibrational analysis. In order to explore the number of apex atoms on the vibrations of the tip, we have simulated the Raman spectra for the same Ag tip consisting of 16,512 atoms with the apex structures from different clusters: a Ag_{10} cluster with the third layer fixed, a Ag_{20} cluster with the fourth layer fixed and a Ag_{56} cluster with the fifth layer fixed. As shown in Figure 3, the dominant vibrational modes are still preserved, although more peaks are present for larger clusters since more atoms are considered during the vibrational analysis.

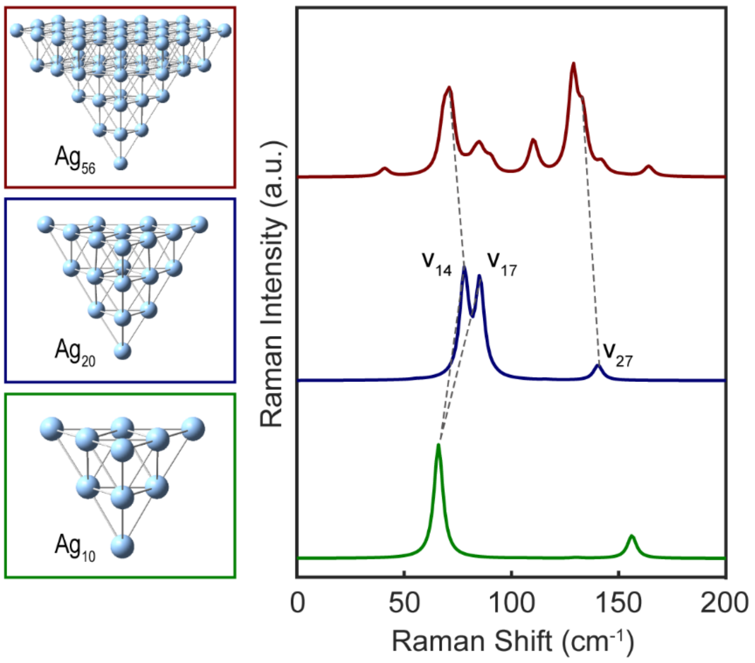

Figure 3. Comparison of simulated Raman spectra of the tip with a height of 15.2 nm and consisting of 16,512 atoms with the apex structures from different clusters: a Ag_{10} cluster with

the third layer fixed, a Ag_{20} cluster with the fourth layer fixed and a Ag_{56} cluster with the fifth layer fixed. The similar vibrational modes are highlighted by the dashed lines.

S5. The apex stacking structures of the Ag tips with different radii

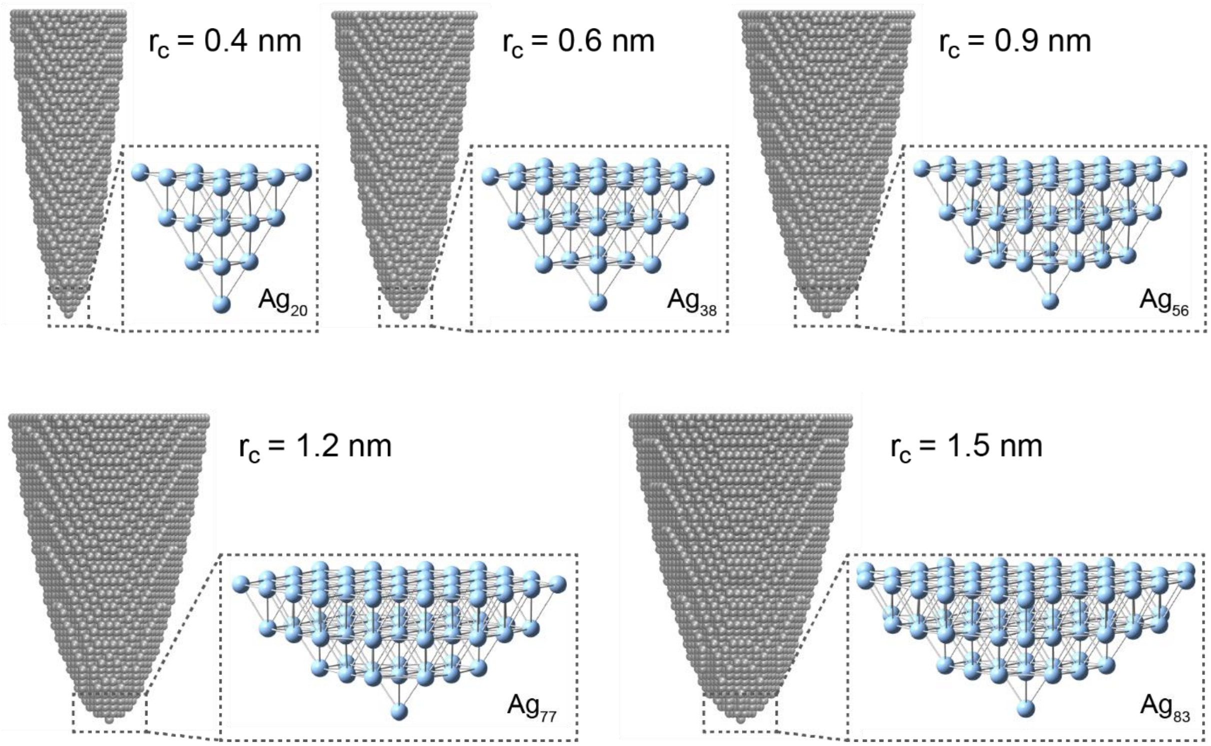

Figure 4. Atomistic stacking structures of Ag tips with different curvature radii, same as the tip structures shown in main-text Figure 5a. The atomic structures of the apex in the gray dashed boxes, as enlarged in the right panels, are used for vibration analysis and the rest atoms in the giant shaft are fixed during all the simulations. The detailed assignment and the schematics of the dominant vibrational modes corresponding to these tips are shown in Video S2 in Supporting Materials.

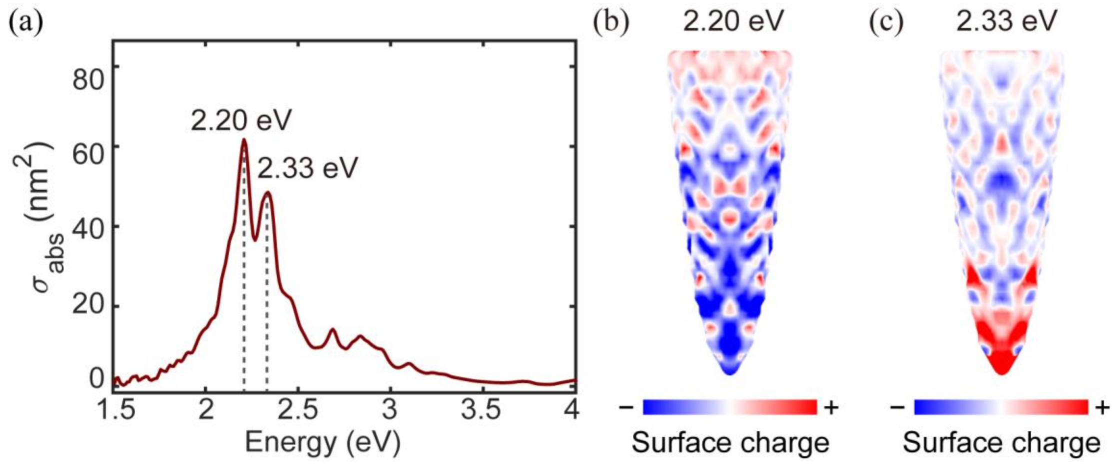

Figure 5. (a) Absorption spectrum and (b-c) the surface charge distribution corresponding to the plasmonic modes at 2.20 eV and 2.33 eV for the tip with a small apex radius of 0.4 nm shown in main-text Figure 5a.

S6. Simulation details of the Raman spectra for large metal tips

In our simulations, the polarizability derivatives are numerically constructed by finite difference method [5] with Equations (8) and (9), in which the induced dipole moments are obtained by solving Equation (2) with either the precise linear solver [6] (abbreviated as "LinSolver") for smaller systems (< 20,000 atoms) or the iterative solver GMRES [7] for large-size tips (> 1,000,000 atoms). Considering different accuracy levels of these two solvers, different displacement step sizes are adopted during the calculations. Here we take the tip with a height of 15.2 nm and a tip radius of 0.8 nm as an example to demonstrate the influence of differential accuracy levels. As shown in Figure 6, if we use the precise solution with LinSolver, the simulated Raman spectra converge once the displacement step size $\Delta\xi_n$ is smaller than 0.1 Å. Therefore, in our simulations, 0.01 Å is selected as the displacement step size for LinSolver. However, for the iterative solver GMRES with a tolerance of 10^{-3}, the Raman intensities are found to decrease if

the displacement step size $\Delta\xi_n$ is smaller than 0.03 Å due to the solution error, thus the step size of 0.1 Å is selected for the simulations based on GMRES.

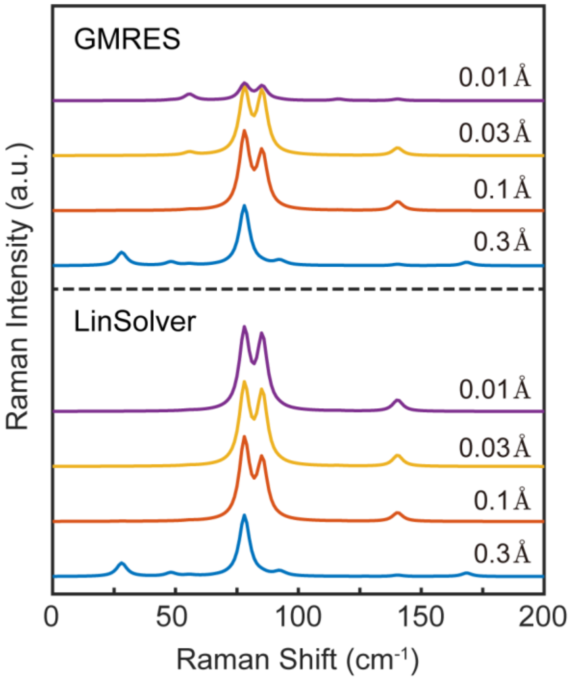

Figure 6. Comparison of the simulated Raman spectra with the absolute Raman intensities for the Ag tip with a height of 15.2 nm and a tip radius of 0.8 nm with different displacement step sizes by using the precise linear solver (LinSolver) and the iterative solver (GMRES), respectively.

For the large-size tip with tens of thousands of atoms, a cut-off condition can be introduced to construct the sparse matrix for the dipole-dipole interaction tensor **T**, ranging from 10^{-6} Å^{-3} for the atoms near the tip apex to 10^{-4} Å^{-3} for the atoms far from the tip apex adapted for different matrix elements following the function

$$T_c = \begin{cases} 10^{-6}, & 1 \le L < 4 \\ 10^{-6} \times 10^{(L-3)/L_r}, & 4 \le L < 2L_r + 4 , \\ 10^{-4}, & L \ge 2L_r + 4 \end{cases} \quad (2)$$

where L represents the layer index where the atom is located and increases from the tip apex (L = 1) to the tip shaft, and L_r represents the rate parameter to tune the cut-off values. The larger the value of L_r, the lower the sparsity of the interaction matrix, indicating the more accurate the Raman spectra. As shown in Figure 7, for the tip with a height of 15.2 nm and an apex radius of 0.8 nm (16,512 atoms, the same tip used in Figure 6), the Raman spectra obtained by solving the sparse matrix (middle panel in Figure 7) with $L_r \geq 30$ are converged to the precisely simulated Raman spectrum obtained by solving the full matrix (top panel in Figure 7). Such convergence also appears in the simulations of the Raman spectra for the larger tip with a height of 50.2 nm and an apex radius of 0.8 nm (183,419 atoms, bottom panel in Figure 7). Therefore, the sparse matrix with the set value $L_r = 30$ is adopted during our simulations of the Raman spectra for large systems.

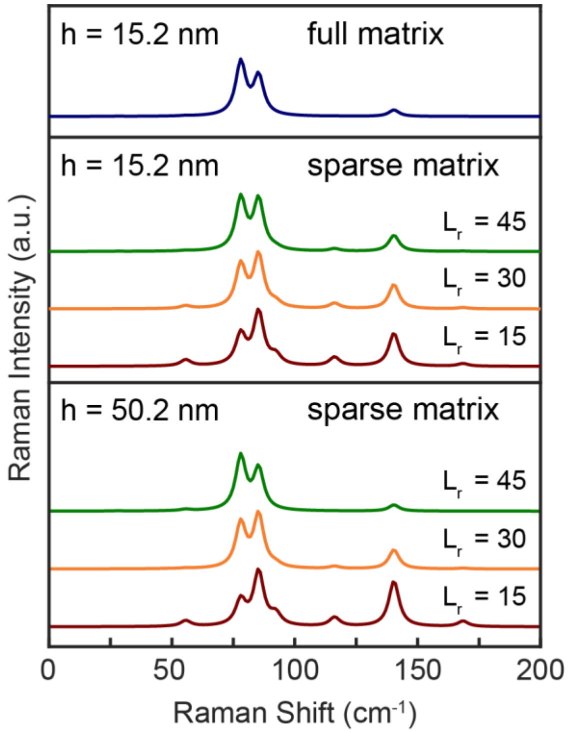

Figure 7. Comparison of the simulated Raman spectra of the Ag tip with a height of 15.2 nm and a tip radius of 0.8 nm (64 layers, 16,512 atoms) using the full matrix (top panel) and the sparse

matrix (middle panel) with different L_r values, respectively. The iterative GMRES is used as the solver for both the sparse and full matrices. The larger tip with a height of 50.2 nm and an apex radius of 0.8 nm (212 layers, 183,419 atoms) is also used as a model system for the test of convergence.

S7. Comparison of the continuous method and the atomistic DDA method

In order to validate the plasmonic response calculated with the atomistic DDA method, a simulation of the local electric field distribution using the finite element method with COMSOL Multiphysics® software are implemented for the same Ag tip with sub-wavelength in main-text Figure 7 and under the same polarization (along z-axis) and wavelength (532 nm) of the incident field. As shown in Figure 8, both methods exhibit very similar distributions of localized field enhancement along the tip surface, with the propagating plasmons more clearly visible in the DDA simulation.

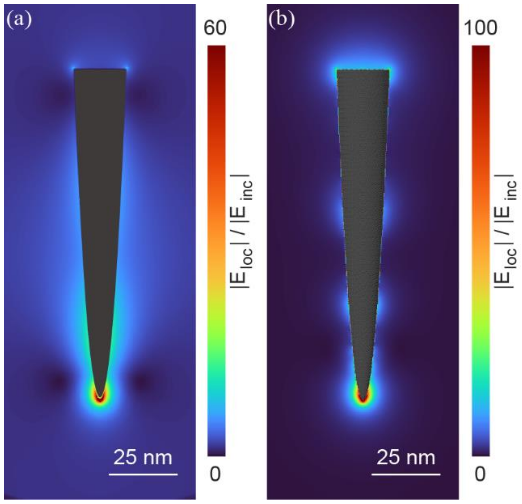

Figure 8. Comparison of local electric field distributions respectively by (a) finite element method and (b) atomistic DDA method, for the Ag tip with sub-wavelength in main-text Figure 7 with the polarization along z-axis and wavelength of the incident field of 532 nm.

REFERENCES

[1] X. B. Zhang, Y. F. Zhang, H. Li, J. Cui, and S. Jiang, "Fast Fabrication and Judgement of Tip-Enhanced Raman SpectroscopyActive Tips," *Chin. J. Chem. Phys.,* vol. 35, no. 5, pp. 713-719, 2022.

[2] F. Benz *et al.*, "Single-molecule optomechanics in “picocavities”," *Science,* vol. 354, no. 6313, pp. 726-729, 2016.

[3] J. Lee, K. T. Crampton, N. Tallarida, and V. A. Apkarian, "Visualizing vibrational normal modes of a single molecule with atomically confined light," *Nature,* vol. 568, no. 7750, pp. 78-82, 2019.

[4] Y. Zhang *et al.*, "Visually constructing the chemical structure of a single molecule by scanning Raman picoscopy," *Natl. Sci. Rev.,* vol. 6, no. 6, pp. 1169-1175, 2019.

[5] B. Fornberg, "Generation of finite difference formulas on arbitrarily spaced grids," *Math. Comput.,* vol. 51, pp. 699-706, 1988.

[6] P. Virtanen, R. Gommers, T. E. Oliphant, et al., "SciPy 1.0: fundamental algorithms for scientific computing in Python," Nat. Methods, vol. 17, no. 3, pp. 261-272, 2020

[7] Y. Saad and M. H. Schultz, "Gmres - a Generalized Minimal Residual Algorithm for Solving Nonsymmetric Linear-Systems," *Siam. J. Sci. Stat. Comput.,* vol. 7, no. 3, pp. 856-869, 1986.
